# Understanding Wellbeing Profiles According to White Matter Structural Connectivity Sub-types in Early Adolescents: The First Hundred Brains Cohort from the Longitudinal Adolescent Brain Study

**DOI:** 10.1007/s10964-024-01939-2

**Published:** 2024-01-13

**Authors:** Christina Driver, Amanda Boyes, Abdalla Z. Mohamed, Jacob M. Levenstein, Marcella Parker, Daniel F. Hermens

**Affiliations:** Thompson Institute, University of the Sunshine Coast, 12 Innovation Parkway, Birtinya, Sunshine Coast, QLD Australia

**Keywords:** Adolescence, Mental health, Wellbeing, White matter

## Abstract

Wellbeing is protective against the emergence of psychopathology. Neurobiological markers associated with mental wellbeing during adolescence are important to understand. Limited research has examined neural networks (white matter tracts) and mental wellbeing in early adolescence specifically. A cross-sectional diffusion tensor imaging analysis approach was conducted, from the Longitudinal Adolescent Brain study, First Hundred Brains cohort (*N* = 99; 46.5% female; *M*_age_ = 13.01, SD = 0.55). Participants completed self-report measures including wellbeing, quality-of-life, and psychological distress. Potential neurobiological profiles using fractional anisotropy, axial, and radial diffusivity were determined via a whole brain voxel-wise approach, and hierarchical cluster analysis of fractional anisotropy values, obtained from 21 major white matter tracts. Three cluster groups with significantly different neurobiological profiles were distinguished. No significant differences were found between the three cluster groups and measures of wellbeing, but two left lateralized significant associations between white matter tracts and wellbeing measures were found. These results provide preliminary evidence for potential neurobiological markers of mental health and wellbeing in early adolescence and should be tracked longitudinally to provide more detailed and robust findings.

## Introduction

Wellbeing is not merely an absence of mental ill health (Ryan & Deci, 2001), with evidence demonstrating that environmental, genetic, and biological markers that delineate mental health, are distinct from those that relate to mental *ill* health (Gatt et al., 2014; Winefield et al., 2012). For adolescents, wellbeing has been found to be a protective factor against the onset of psychopathology (Campbell & Osborn, 2021), which is important, as 50% of mental disorders occur before the age of 14 and 75% before 24 years old (Kessler et al., 2005). Adolescence; defined as the transition between childhood and adulthood (Sawyer et al., 2018), is also a time for significant neurodevelopment, with such changes occurring in combination with psychological, cognitive and behavioral maturation (Lebel et al., 2019). From a neurodevelopmental perspective, adolescence is marked by a period of heightened neuroplasticity, but also vulnerability, and can be affected by experiences and environmental factors (Fuhrmann et al., 2015). Such factors can potentially affect short and long-term mental health and wellbeing either positively or negatively (Aoki et al., 2017). Studies have highlighted the associations between adolescent neurodevelopment and psychopathology, or mental ill health (e.g., (Fuhrmann et al., 2015)), yet only recently has there been a shift in focus to understand the neural correlates of wellbeing in “general” adolescent populations. Thus, elucidating the neural indicators of mental wellbeing as young people commence adolescence may be as important as understanding the neural markers of mental ill health. More specifically, it has been suggested that there is a need to clarify if, and which, neural networks may be altered with the presence, or absence of, wellbeing and resilience (Gatt et al., 2018). The current study addresses these gaps by using an exploratory data-driven approach, aiming to characterize potential neurobiological profiles in early adolescence, which may be associated with mental health and wellbeing.

### Wellbeing

Wellbeing has been described as a multidimensional phenomenon comprising of hedonic (subjective), and eudemonic (psychological) wellbeing (Ryan & Deci, 2001). Subjective wellbeing reflects the momentary experience of pleasure, with specific regard to experiencing positive affect, a lack of negative affect, and current life satisfaction (Ryff & Keyes, 1995). Psychological wellbeing relates to human potential, specifically autonomy, life purpose, mastery, personal growth, positive relatedness, and self-acceptance (Ryff & Keyes, 1995). Although early research suggested that across the lifespan, pursuing pleasurable activities and maintaining relationships was central to wellbeing (Ryff, 1989), recent research suggested that younger adults and adolescents place more emphasis on subjective wellbeing, such as experience expansion, social networks, knowledge and success, and novelty seeking (thrill and happiness “for me”), than other ages (Karwetzky et al., 2021). In a study of almost 10,000 early adolescents (aged 11–14 years old), the unstable nature of subjective wellbeing (life-satisfaction) over time was reported, with females reported to be more prone to reductions in wellbeing (Patalay & Fitzsimons, 2018). Alternatively, subjective wellbeing (health related quality of life) in adolescents (aged 12–15 years old at baseline) was found to remain largely stable over time, but sex differences were reported, with females reporting lower wellbeing overall (Meade & Dowswell, 2016). Whilst both studies represent aspects of subjective wellbeing, in a study with a sample of *N* = 747 adolescents (aged 13–17 years), psychological wellbeing dimensions of self-acceptance, autonomy, and life development, were reported to remain stable over time in both males and females, with only the positive interpersonal relationships dimension showing an increase over the 8 month period (Gómez-López et al., 2019). Although such research sheds a light on subjective wellbeing, and psychological wellbeing independently, the concept of optimal wellbeing has long been recognized as encompassing both subjective and psychological wellbeing, and therefore is important for prevention and intervention programs in adolescence to target both domains (Tejada-Gallardo et al., 2020). Further, due to the reported shared variance between subjective wellbeing, and psychological wellbeing domains, it is imperative to consider an aggregate wellbeing measure (Gatt et al., 2018). Taken together, these results suggest that there is a need to further understand wellbeing in early adolescence using a composite measure of overall wellbeing, along with subjective and psychological wellbeing independently.

Optimal wellbeing was once considered to be on a single bipolar continuum with mental ill health at the other extreme. However, research now evidences that although psychopathology and wellbeing are consistently associated, they operate on a functionally independent dual continua, and as such, it can be postulated that wellbeing can be enhanced despite the occurrence of mental ill health (Keyes, 2005; Mason Stephens et al., 2023). Further, evidence suggests that measuring one construct alone, may not be sufficient for understanding and interpreting levels of the other, and unidimensional scales for psychopathology and wellbeing may result in inaccurate interpretations of overall wellbeing (Mason Stephens et al., 2023). The dual-factor model therefore emphasizes the importance of considering both psychopathology *and* wellbeing (Keyes, 2005), and in early adolescence specifically, the dual-factor model has been found to be useful in observing and understanding changes in psychopathology and wellbeing profiles (Petersen et al., 2022). Consequently, there is merit in measuring various components of wellbeing and psychopathology to further understanding of adolescent mental health.

### Adolescent Brain Development

Complex patterns of change in white matter and gray matter occur throughout the lifespan (Fuhrmann et al., 2015), and in adolescence this follows a typical pattern of gray matter volume decrease and white matter volume increase (Giedd, 2004), in a posterior/inferior to anterior direction (Olson et al., 2015). Typically, the beginning of adolescence is defined as being around 12 years of age coinciding with the commencement of secondary school, as well as being around the time of pubertal onset. In line with early adolescence, myelination of the brain’s anterior regions commences, and continues into early adulthood (Blakemore & Choudhury, 2006). However, this process is non-linear (Lebel et al., 2019), and the adolescent years are a particularly sensitive period for myelination of anterior brain regions (Olson et al., 2015). The rate of white matter maturation is thought to vary regionally throughout the adolescent brain, with maturation in association tracts that comprise frontal-temporal connections following a more protracted developmental period compared to commissural or projection tracts (Lebel et al., 2019). Literature regarding typical white matter development from childhood to young adulthood has been mixed, with some evidence suggesting regionally dependent sexual dimorphism in white matter in adolescence, yet other evidence pointing to no differences in sex, and pubertal effects on white matter trajectories remain unclear (Lebel et al., 2019). Further it appears that many cross-sectional studies on “adolescent” white matter, comprise of participants covering a broad range of ages from early childhood (aged 4 years) up to young adulthood (aged 30 years) (Lebel et al., 2019), which may limit generalizability of findings, particularly when investigating different phases of adolescence (e.g., early adolescence compared to late adolescence). Hence, studies that focus on narrower age ranges within the adolescent period are needed.

### Diffusion Tensor Imaging

Studies utilizing a diffusion tensor imaging (DTI) approach can examine the structure of white matter tracts in vivo, through measurement of water diffusivity. DTI is a non-invasive Magnetic Resonance Imaging (MRI) technique that quantifies the diffusion distribution of water within each voxel along three orthogonal axes. Fractional anisotropy (FA), the most reported DTI metric, measures diffusion directionality with a range from 0 (isotropic diffusion—equal in each direction) to 1 (diffusion occurs in one direction). Axial diffusivity (AD) refers to the magnitude of diffusion in the principal direction of diffusion, whereas radial diffusivity (RD) refers to diffusion perpendicular to the principal diffusion direction on the secondary and tertiary axes. Mean diffusivity is calculated from the averaged diffusion along all three axes. FA values may be used as proxy measurements of white matter tract integrity, encompassing axon diameter and relative alignment, axonal density, and thickness of the myelin sheath (Beaulieu, 2002). Abnormally low FA values may signify loss of axons and/or demyelination, whereas it may be inferred from abnormally high FA that there may be dense axonal packing, excessive myelination, and/or reduced neural branching and functionality of the myelin sheath (Soares et al., 2013). Mean diffusivity is also sensitive to the aforementioned indicators of white matter integrity, but RD and AD values are thought to provide more specific measures of white matter microstructure compared to FA or mean diffusivity alone (Goddings et al., 2021). Specifically, AD is a more explicit marker of axon numbers and axonal coherence, whereas RD is more sensitive to changes in myelination, axonal packing or myelin integrity (Lebel et al., 2019). Studies in healthy children also suggest that FA and RD values represent axonal packing and diameter and/or myelination (Krogsrud et al., 2016), and AD values in late adolescence and adulthood may represent axon straightening (Giorgio et al., 2010), with many adolescent brain studies reporting that increases in FA and decreases in mean diffusivity are largely driven by decreasing RD (Lebel et al., 2019), reflecting large axonal diameter, myelination and dense axonal packing (Feldman et al., 2010).

DTI analysis is widely utilized in the literature to infer the relationships between white matter structure and integrity, and behavioral, psychological, and cognitive measures (Goddings et al., 2021). Historically the focus of the connection between white matter development and cognitive and behavioral changes has been on markers of specific mental disorders as opposed to markers of mental health and wellbeing. For example, FA irregularities have consistently been found across studies examining numerous mental disorders, including affective disorders (Sexton et al., 2009), bipolar disorder (Heng et al., 2010), psychotic disorders (Hermens et al., 2019), and schizophrenia specifically (Kanaan et al., 2005), yet such abnormalities are not always specific to an individual disorder and findings vary considerably between studies. Further, it is an equally important aim to understand the neurobiological profiles that may indicate, or be associated with, adolescent mental health and wellbeing.

### Wellbeing and the Adolescent Brain

Research into the associations between neural indicators and various components of wellbeing, had previously been dominated by adult population studies, with a focus on gray matter volume in regions typically associated with emotion or reward processing (Gatt et al., 2018). In adolescents, several recent volumetric gray matter studies have investigated mental health and wellbeing. For example, in one of the first studies in a community sample of early to mid-adolescents aged 12–17 years (*N* = 89), results suggested that more positive affect (subjective wellbeing) resulted in greater decreases in caudate volume over time, and positive affect was also found to be associated with larger hippocampal volume (Dennison et al., 2014). Similarly, using the COMPAS-W (Gatt et al., 2014), which includes measures of subjective, and psychological wellbeing and composite wellbeing scores, a previous study from the Longitudinal Adolescent Brain Study (Boyes et al., 2022) found negative associations between left caudate volume and scores on “composure” and “positivity” (both subjective wellbeing, and closely related to optimism and positive affect), and “total wellbeing”, in 12-year old’s indicating that smaller caudate volume at age 12 is linked to increased subjective wellbeing.

Numerous studies have investigated functional and connective neural correlates of various aspects of wellbeing, such as eudemonic wellbeing (Kong et al., 2015), flourishing (Goldbeck et al., 2019), happiness (Luo et al., 2016) and subjective wellbeing (Shi et al., 2018), implicating several regions associated with the default mode and salience networks. However, results across studies have been largely heterogenous in terms of wellbeing measures and associated brain activity, with study samples comprised of mostly young adult and/or adults, as opposed to early adolescents exclusively.

Studies specifically investigating white matter and how it may be linked to aspects of mental health and wellbeing have been limited until recently. Vitolo et al. (2022) measured emotion regulation and white matter microstructure, finding anomalies across tracts linking occipital, parietal, and temporal regions, whereby those with low re-appraisal had increased mean diffusivity in clusters within the superior longitudinal fasciculus (SLF), superior corona radiata (SCR) and anterior uncinate fasciculus (UF). Kotikalapudi et al. (2022) investigated personality profiles that may be predictive of wellbeing and the potential relationships with white matter. They found that optimism was positively associated with AD values exclusively in the right hemisphere, and specifically within the anterior cingulum bundle (CNG), anterior corpus callosum (CC genu), anterior thalamic radiation (ATR), corticospinal tract (CST—which had the strongest finding relating to cluster size), SLF, and UF. However, both studies did not directly measure wellbeing, as Vitolo et al. (2022) measured emotion regulation, while Kotikalapudi et al. (2022) assessed personality profiles, that may predict wellbeing; and both studies had mostly or only female samples aged from 18 to 40 years. Further, in a multi-modal study, the associations between gray matter volume, white matter microstructure, and subjective wellbeing in a cohort of 17–65 year old’s was investigated (Jung et al., 2022). Findings revealed that higher levels of subjective wellbeing were associated with increased gray matter volumes of the anterior insula, as well as decreased FA values in clusters of the CC body, fornix cres/stria terminalis (FCST) and precuneus white matter. Further, negative correlations were found between quality of life and FA values in the CC body, FCST and precuneus white matter, and between positive emotion subscale scores, and the posterior corona radiata (PCR), SLF, and CC splenium. Jung et al. (2022) concluded that higher subjective wellbeing may be characterized by decreased connectivity in the FCST and CC body. However, their study sample comprised of participants aged 17–65 years, only measured subjective wellbeing and reported FA values as the only measure of white matter microstructure. Given the varied and somewhat disparate findings relating to white matter and mental health and wellbeing in early adolescence, it is speculative at this stage to postulate any independent neurobiological mechanisms for these different aspects of wellbeing. Therefore, it is imperative to provide additional evidence to build a more comprehensive understanding of the neurobiology of wellbeing in adolescence.

## Current Study

Despite prior research investigating the neural correlates of mental health and wellbeing, significant gaps in knowledge still exist, especially in early adolescence. Much of the aforementioned research used either a measure of subjective wellbeing or psychological wellbeing, but not both, and according to the dual factor model, whilst psychopathology and wellbeing operate on a functionally independent dual continua, it is important to consider both psychopathology and various domains of wellbeing. Further, evidence suggests that measuring one construct alone, may not be sufficient for understanding and interpreting levels of the other, and unidimensional scales for psychopathology and wellbeing may result in inaccurate interpretations of overall wellbeing (Mason Stephens et al., 2023). Therefore, this study used a composite measure of wellbeing encompassing both subjective wellbeing and psychological wellbeing, a quality-of-life measure, and a psychological distress measure. Based on the literature, 21 white matter tracts were chosen as regions of interest representing the three major types: (i) association fibers (SLF, UF); connect cortical areas within each hemisphere, (ii) projection fibers (PCR, SCR, CST, PTR, FCST, PLIC, CNG); connecting cortical areas with subcortical structures, and (iii) commissural fibers (CC genu, CC body, CC splenium); connecting similar cortical areas between hemispheres. Given the heterogeneous findings across previous DTI studies in adolescent populations, and the need to consider both individual and collective measures of mental wellbeing, an exploratory data-driven approach was undertaken. Additionally, utilizing cluster analysis as an optimal method for characterizing potential neurobiological profiles, this study aims to inform future research around potential associations between mental health and wellbeing measures and white matter profiles in early adolescence.

## Methods

Ethics approval was granted by the University of the Sunshine Coast Human Research Ethics Committee (HREC# A181064). Informed, written consent was obtained from both the parent/caregiver and the adolescent prior to participation in the study.

### Participants

Participant data was acquired as part of the Longitudinal Adolescent Brain Study (LABS), Australia. Participants came from a self-selected community derived sample, who were recruited from the Sunshine Coast area via a range of networks, including local media, community services, and schools. The LABS aims to track (via neuroimaging and neuropsychological assessments) longitudinal changes in the adolescent brain at four-monthly intervals over a period of 5 years. Data from the LABS general population First Hundred Brains cohort (see Levenstein et al., 2023) were utilized for this study (*N* = 101; 46.5% female). Participants were between the ages of 12 and 13 years old (*M*_age_ = 13.01, SD = 0.55), and proficient in spoken and written English. Exclusion criteria included individuals who reported having a major neurological disorder, intellectual disability, medical illness, or those who reported sustaining a head injury that involved loss of consciousness for greater than 30 min. The methodology, selection, and inclusion criteria for the First Hundred Brains cohort specifically, are outlined in detail elsewhere (see Levenstein et al., 2023).

### Procedure

For this specific study, data was used from the First Hundred Brains cohort of participants who completed self-report questionnaires including; the COMPAS-W (Gatt et al., 2014), Kessler Psychological Distress scale (K10) (Kessler et al., 2002), WHO-QOL BREF (Whoqol, & Group, 1998), as well as those who had undergone MRI, including DWI scans. A whole-brain DTI technique was employed to determine FA values. Next, cluster analysis was performed utilizing FA values obtained from key white matter tracts. Cluster analysis is a hypothesis generating technique, therefore, to better understand the functional implications of the cluster groups identified, the association between FA and self-report measures were also examined.

### Measures

#### Wellbeing (COMPAS-W)

As part of the self-report questionnaire, participants completed the 26-item COMPAS-W wellbeing measure to assess Total Wellbeing, which is a reliable indicator of mental health, as well as wellbeing subscales: Composure, Own-worth, Mastery, Positivity, Achievement and Satisfaction, to identify specific areas of strength and deficit (Gatt et al., 2014). Each item is scored using a 5-point Likert-type scale (1 = *strongly disagree* to 5 = *strongly agree*) with a potential “total wellbeing” score ranging from 26 to 130. Six subscales represent a unique wellbeing construct and include composure, own-worth, mastery, positivity, achievement and satisfaction. Summing of individual items results in scores ranging from a minimum of 3 to a maximum of 9, with some items loaded on multiple subscales. Validated for use with 12–61 year-olds, the COMPAS-W includes measures of eudemonic (psychological wellbeing; own-worth, mastery and achievement), and hedonic (subjective wellbeing; composure, positivity and satisfaction) (Gatt et al., 2020). COMPAS-W has recently been utilized in research linking wellbeing to subcortical gray matter volume in adults (Gatt et al., 2018), and in 12 year old’s (Boyes et al., 2022). In the current sample, there was good internal consistency (Cronbach’s α = 0.856). Cut-offs for groups were based on the categories established by Gatt et al. (2014), whereby “total wellbeing” scores of 89 or below were classified as “languishing”, 90–110 “moderate”, and 111 or higher were classified as “flourishing”. The COMPAS-W can therefore be used as a continuous variable or using categorizations. As mental illnesses can be diagnosed based on symptoms and analyses of functioning, individuals can also be placed on a “wellbeing continuum”. “Languishing” wellbeing in adults has been characterized by impairments to psychological, social, and physical factors, and an increased likelihood of a depressive episode (Keyes, 2002). By contrast, those who experience “moderate” or “flourishing” wellbeing, appear to function better in terms of relationships, educational attainment, and daily living, with most people falling in the “moderate” category (Keyes, 2002). However, it should not be assumed that all individuals who are experiencing symptoms of a depressive episode are “languishing”, and further, “languishing” individuals who are not depressed, can experience greater impacts to their daily life (Keyes, 2002).

#### Psychological Distress

The K10 (Kessler et al., 2002) is a 10-item self-report questionnaire pertaining to non-specific symptoms of psychological distress, requiring respondents to report how often they have experienced such symptoms over the past 30 days. Responses are rated on a 5-point Likert scale ranging from 1 = *None of the time* to 5 = *All of the time* on items such as “*during the last 30 days, about how often did you feel worthless”*. Scores range from 10 (indicating low levels of distress) to 50 (indicating severe levels of distress). The K10 has been used in several prominent Australian and international health surveys, inclusive of the Australian Health Survey, the New Zealand Health Survey, and the Canadian Community Survey, and has been validated for use in adolescents aged 12 to 19 years *N* = 2325 (Chan & Fung, 2014). In the current sample, the K10 had good internal consistency (Cronbach’s α = 0.871). The K10 can be used as a continuous variable, or scores can be categorized into groups. Cut-offs for groups were based on the Australian Bureau of Statistics Information Paper which categorizes K10 scores of 10–15 as “low” (likely to be well), 16–21 “moderate”, 22–29 “high” and 30–50 “very high” (Andrews & Slade, 2001; Australian Bureau of Statistics, 2012). Although high scores on the K10 are strongly associated with depressive or anxiety disorders, the experience of psychological distress does not classify someone as experiencing mental disorder (Andrews & Slade, 2001). Nonetheless, high and very high levels of psychological distress are suggested to be a proxy measure for the presence of mental ill health (Australian Institute of Health & Welfare, 2021). The K10 scale was utilized as an additional measure to the COMPAS-W, to delineate those who scored low on psychological distress and were therefore categorized as “well” as per the ABS categories (Andrews & Slade, 2001; Australian Bureau of Statistics, 2012).

#### Quality of Life

The World Health Organization Quality of Life (WHOQOL-BREF) (23 items) measures the following broad domains: (i) physical health, referring to energy, fatigue, pain, discomfort, sleep, and rest; (ii) psychological health, referring to bodily acceptance, negative and positive feelings, self-esteem, and cognition; (iii) social relationships, referring to personal relationships and social support; (iv) environment, referring to financial resources, freedom, physical safety and security, health and social care accessibility and quality, home environment, opportunities for acquiring new information and skills, and physical environment. Total scores are achieved for each domain, measured on a scale of 1 to 5, with higher scores indicating better quality of life in that domain. The WHOQOL-BREF has been employed extensively by both psychological and medical research as a cross cultural measure of health-related quality of life (Chen et al., 2006), and has been validated in a sample of 365 Taiwanese, high school students aged between 12.58 and 14.78 years (Chen et al., 2006). In the current sample, this measure had good internal consistency (Cronbach’s α = 0.918). The WHO QOL BREF was used as an additional measure with reference to quality of life and life satisfaction as a marker of mental health and wellbeing.

### MRI Acquisition

The MRI data were collected using Siemens Skyra 3 T MRI scanner using a 64-channel head and neck coil at the XXX Institute. The structural 3D T1-weighted images were collected using magnetization prepared-rapid gradient echo (MP-RAGE) sequence with TR/TE/flip angle = 2200 ms/1.76 ms; matrix = 256 × 256 × 208, and resolution = 1.0 × 1.0 × 1.0 mm. The total time for the MP-RAGE acquisition was 4 min. The diffusion tensor imaging data were acquired using a multi-slice spin echo-planner imaging sequence with TR/TE = 3300 ms/115 ms, matrix = 114 × 114 × 72, resolution = 2.0 × 2.0 × 2.0 mm^3^, and b-values = 8 × b = 0 s/mm^2^ + 27 directions with *b* = 1000 s/mm^2^ + 62 directions with *b* = 2500 s/mm^2^. A reversed phase encoding DTI data with 6 × b = 0 s/mm^2^ were also acquired for EPI distortion correction during data pre-processing. The total time for the DTI acquisition was 10 min.

### MRI Data Pre-Processing

Data were pre-processed and analysed using FMRIB’s Software Library (FSL 5.0.9) as well as MRtrix 3.0 (Tournier et al., 2019) and Advanced Normalization Tools (ANTs; 2.0.1; https://www.nitrc.org/projects/ants). DTI data were denoised using MRtrix-dwidenoise (Cordero-Grande et al., 2019) which estimates the noise level and denoises the data based on random matrix theory as it exploits data redundancy in the patch-level PCA domain. The b_0_ images of the forward and reverse phase acquired DTI images were prepared as input for the FSL-Topup (Andersson et al., 2003) function for susceptibility distortion correction, then were corrected for eddy current using (FSL-eddy_openmp). Data were also corrected for field bias using MRtrix-dwibiascorrect (Tustison et al., 2010), and the mask was estimated using MRtrix-dwi2mask. To estimate the DTI matrices, MRtrix dwi2tensor was used to compute the corresponding tensor components, then MRtrix-tensor2metric was used to generate the eigenvector maps used to estimate the FA, RD and AD.

After pre-processing, out of the 101 participants included in the First Hundred Brains cohort two participants were excluded because of incomplete data acquisition (missing MRI data) resulting in a sample size for the current study, of *N* = 99. To normalize the data to a common space, and as the sample were young adolescents, a study specific template was created. To generate the template, the FA of individual maps were linearly registered to the FMRIB58 template-space using FSL’s-FLIRT with 6 degrees of freedom, then all individual maps were averaged to generate the initial template (i.e., Template0). Then a study specific template was generated using the antsMultivariateTemplateConstruction2.sh script from ANTS (Avants et al., 2011). Once all the FA maps were non-linearly registered to the study-specific template, the transformation and warp files were used to register the RD, and AD maps to the study specific template. The group mean FA images were generated and then skeletonized to identify the centers of white matter tracts with a threshold FA value of 0.2. The Johns Hopkins University - International Consortium for Brain Mapping (JHU-ICBM) DTI 81 labels atlas (Mori et al., 2005) as part of FSL was used to create tract specific masks of the tracts of interest and to extract the mean values of the DTI measures from different white matter tracts. A total of 21 tracts were used in this analysis.

### Statistical Analyses

Statistical analyses were conducted using the Statistical Package for Social Sciences (SPSS version 26). Means, standard deviations, and frequencies of demographic and self-report measures for the whole sample were first calculated. Next, independent sample *t* tests (with Levene’s test) and chi-squared tests were performed to identify any significant differences between males and females on the self-report measures scores and categories, and age. Prior to the cluster analysis, the DTI variables were checked for multicollinearity. For the cluster analysis, mean FA scores for each of the 21 white matter tracts were standardized (converted to z-scores) across the sample of *N* = 99, so that they could be comparable. Next a hierarchical cluster analysis with Wards method of minimum variance with a squared Euclidean distance measure was performed. Cluster analysis creates homogenous groups from the data and was therefore utilized in order to initially identify FA profiles across the sample. Discriminant function analysis was conducted (standard, confirmatory) to determine which combination of FA variables best distinguished the cluster groups.

Differences in DTI (FA, AD, RD), demographic data, COMPAS-W scores, subscales, and categories, K10 scores and categories, and WHO-QOL scores and subscales across the cluster groups were assessed using one-way analysis of variance (ANOVA) or chi-squared tests. For the ANOVAs, Scheffe’s tests were used to determine post hoc pairwise comparisons of cluster groups, and for the Chi-squared tests, adjusted standardized residuals were analysed and converted to *p* values, which were subsequently adjusted using a Bonferroni correction. The significance level for the above-mentioned tests was *p* < 0.05, except for the Chi-squared residuals post hoc test, where a Bonferroni correction for multiple comparisons was applied accordingly (adjusted *p* < 0.0042).

To identify which features of diffusivity (i.e., AD/RD) may best describe any differences among the clusters, a multinomial logistic regression analysis was performed. The dependent variable was the cluster group, with RD and AD scores as co-variates. To achieve a parsimonious model, the four tracts that best differentiated the clusters were chosen. AD and RD scores of the tracts were standardized to create a comparable scale and were checked for collinearity. Any variables (apart from FA values) that were significantly different between cluster groups were to be included in the model as covariates or factors. If the goodness of fit statistics were significant and evidence of over dispersion was present, a standard error correction was performed with the appropriate dispersion parameter.

Pearson’s correlations were conducted to examine associations between key FA values for each tract and self-report measures for the whole sample, as well as within each cluster group. Given the number of correlations carried out, a Bonferroni correction was applied for each set of correlations, with the resultant significance level set at *p* < 0.0016.

### Data Screening

Data cleaning and examination of self-report data and DTI metrics showed that all outcome variables comprised valid scores (no missing data) and were then assessed for normality and outliers. Using Shapiro-Wilks’s test of normality, COMPAS-W total wellbeing scores (and subscales), K10 scores and WHOQOL total scores (and subscales) were deemed non-normal (*p* < 0.05). However, visual inspection of the histograms and box plots showed relatively normal distributions for all scales and subscales with no outliers. The only exception was positivity, which was slightly skewed towards higher scores (skewness = −0.91). However central limit theorem offers protection for violation of normality if the sample is over *N* = 30 for all dependent variables, therefore no transformations were performed. All other variables (DTI metrics) were normally distributed.

## Results

### Whole Sample

Data from *N* = 99 participants (*M*_age_ = 13.01, *SD* = 0.55) were accessed as part of LABS, with demographics and variables scores for the whole sampled outlined in Table 1. There were no significant differences between males and females for age, total wellbeing scores, psychological distress categories, wellbeing categories, and all wellbeing sub-scale scores, except mastery. Females had significantly higher mastery scores (*M* = 23.46, *SD* = 3.61) than males (*M* = 21.91, *SD* = 3.03, *t* = −2.326 [97], *p* = 0.011). The only other significant difference between males and females was for psychological distress scores, where females had higher scores (*M* = 17.30, *SD* = 6.82) than males (*M* = 14.43, *SD* = 4.34, *t* = −2.456 [74.3], *p* = 0.016). This is in line with population data reported in the Young Minds Matter Survey of 11–17 year olds (using the K10 and ABS derived cut-offs that were also used for the current study), where a higher proportion of females had high or very high levels of psychological distress (Australian Institute of Health and Welfare, 2021). Further in the whole sample of the current study (Table 1) 15.1% had high or very high levels of psychological distress which is also comparable with the results from the Young Minds Matter Survey, where it was reported that 16.8% of 11–17-year-olds had high or very high psychological distress.

**Table 1:** Whole sample and cluster demographic characteristics and variable scores

	Whole sample *N* = 99 *M* (SD)	Cluster 1 *n* = 48 *M (*SD)	Cluster 2 *n* = 28 *M* (SD)	Cluster 3 *n* = 23 *M* (SD)
COMPAS-W Total	100.33 (11.18)	100.38 (11.95)	101.21 (8.94)	99.17 (12.30)
K10 Total	15.77 (5.78)	15.48 (6.00)	14.89 (3.12)	17.43 (7.48)
WHO-QOL Total	97.09 (10.37)	97.21 (10.94)	98.04 (9.45)	95.70 (10.48)
SWB (hedonic)				
Composure	14.42 (2.98)	14.83 (2.87)	14.54 (2.92)	13.34 (3.19)
Positivity	20.98 (2.86)	20.92 (3.03)	21.57 (2.06)	20.39 (3.27)
Satisfaction	35.62 (5.30)	35.69 (5.55)	36.39 (3.67)	34.52 (5.89)
PWB (eudemonic)				
Own worth	33.25 (4.45)	33.21 (4.44)	33.64 (3.62)	32.87 (5.43)
Mastery	22.63 (3.38)	22.65 (3.20)	22.61 (3.65)	22.61 (3.55)
Achievement	11.60 (2.12)	11.33 (2.30)	11.36 (1.59)	12.43 (2.15)
QoL				
Physical	21.08 (2.63)	21.02 (2.77)	21.39 (2.20)	20.83 (2.85)
Psychological	23.74 (3.42)	23.81 (3.41)	24.39 (2.98)	22.78 (3.84)
Social	8.46 (1.36)	8.46 (1.41)	8.61 (1.17)	8.30 (1.49)
Environmental	34.95 (4.03)	35.10 (4.27)	34.86 (3.75)	34.74 (4.01)
Overall	8.86 (1.13)	8.81 (1.07)	8.79 (1.17)	9.04 (1.22)
	Whole sample *N* (%)	Cluster 1 *n* (%)	Cluster 2 *n* (%)	Cluster 3 *n* (%)
Females	46 (46.5)	20 (41.6)	13 (46.4)	13 (56.5)
Wellbeing category				
Flourishing	17 (17.2)	11 (22.9)	3 (10.7)	3 (13.1)
Moderate	63 (63.6)	26 (54.2)	22 (78.5)	15 (65.2)
Languishing	19 (19.2)	11 (22.9)	3 (10.7)	5 (21.7)
Psychological distress category				
Low/Well	60 (60.6)	31 (64.6)	16 (57.1)	13 (56.5)
Moderate	24 (24.2)	9 (8.3)	11 (39.3)	4 (17.4)
High	12 (12.1)	6 (12.5)	1 (3.6)	5 (21.7)
Very high	3 (3.0)	2 (4.2)	0 (0)	1 (4.3)

### Cluster Characteristics

Agglomeration coefficients generated by the cluster analysis for the 21 white matter tracts revealed a demarcation point between three and four cluster solutions. For optimal statistical power, cluster groups should be *n* > 20 (Dalmaijer et al., 2022), thus a three-cluster solution was selected. This was confirmed via inspection of the dendrogram. The resultant cluster groups/sizes were: cluster 1 [*n* = 48]; cluster 2 [*n* = 28], and cluster 3 [*n* = 23] and their demographic and self-report data are provided in Table 1. Among the cluster groups there were no significant differences in wellbeing, quality of life and psychological distress scores (or subscale scores). Further, when evaluating wellbeing and psychological distress categories, Chi squared tests revealed that there were no significant differences between clusters. Finally, there were no significant differences in the proportion of females-to-males among the three cluster groups (see Table 1).

**Fig. 1:**
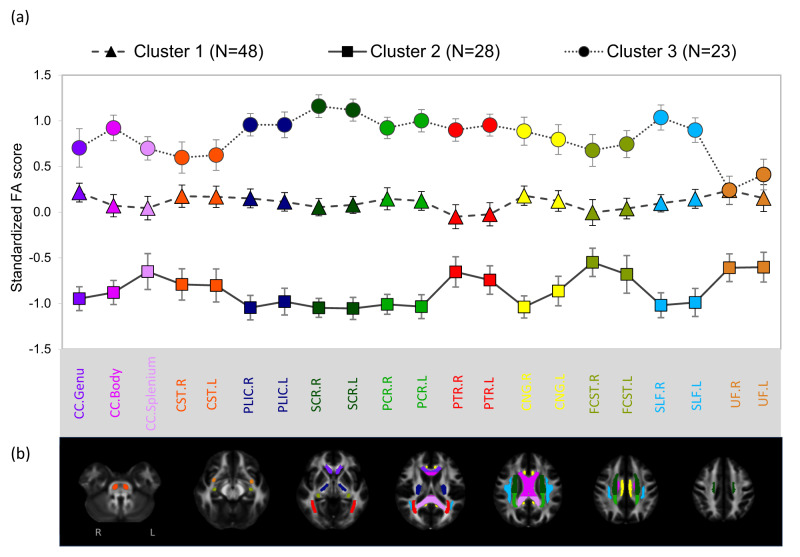
**a** Standardized FA scores (mean with 95% confidence interval bars) across cluster groupings, derived from 21 white matter-tracts (JHU-ICBM atlas). **b** Visualization of the 21 JHU-ICBM atlas tracts included in the cluster analysis, overlaid on a standard space template. Axial slices depict inferior to superior moving from left to right. For greater distinction, bilateralized or single tracts are each color coded uniquely and correspond to the labels on the x-axis in subplot-a

Across the three cluster groups distinct profiles of FA scores were evident (Fig. 1). All 21 of the DTI variables revealed significant main effects of “cluster group” (*p* < 0.001). Specifically, cluster 2 showed significantly decreased FA scores across all tracts compared to the other clusters (Scheffe’s; *ps* ≤ 0.045). In contrast, cluster 3 showed significantly increased FA scores (Scheffe’s; *ps* ≤ 0.017) across all tracts except for the CST-R and L, UF-R and L, and CC genu (no different from cluster 1). Compared to the other clusters, cluster 1 showed an intermediate FA profile.

To evaluate the parameters underlying FA, clusters were also compared in terms of AD and RD. For AD scores, ANOVA revealed significant main effects of “cluster group” in only three of the tracts—the CNG-R & L, and the PLIC-L (*ps* ≤ 0.015). In the CNG-R, cluster 2 had significantly decreased AD scores compared to cluster 1 and 3 (Scheffe’s; *ps* ≤ 0.026). In the CNG-L, cluster 2 had significantly decreased AD scores compared to cluster 3 (Scheffe’s; *ps* ≤ 0.016). In the PLIC-L, cluster 2 had significantly decreased AD scores compared to cluster 1 and 3 (Scheffe’s; *ps* ≤ 0.037). No other significant main effects were found for AD scores.

For RD scores, there were significant main effects of “cluster groups” for all tracts (*ps* ≤ 0.010) except for FCST-R and the CST-R. Specifically, cluster 2 showed significantly increased RD scores in 14 of the tracts compared to the other clusters (Scheffe’s; *ps* ≤ 0.043). The only exceptions were the CST-L, the PTR-R, the PTR-L, and the FCST-L, where cluster 2 had significantly increased RD scores compared to only cluster 3, the UF-R where cluster 2 had significantly increased RD scores compared to only cluster 1 (Scheffe’s; *p* = 0.023)., and in the PTR-R, FCST-L cluster 3 had significantly decreased RD scores compared to only cluster 1 (Scheffe’s; *p* = 0.002).

### Discriminant Function Analysis

With the 21 FA variables entered simultaneously as predictors, discriminant function analysis confirmed the distinct FA profiles, generating two functions to separate the three cluster groups. The first function accounted for 95.5% of the differences among the clusters (Wilk’s λ = 0.119, *p* < 0.001). The second function explained the remaining 4.5% of the variance and was also statistically significant (Wilk’s λ = 0.709, *p* = 0.004). The structure matrix showed a clear delineation, with function 1 being characterized by high to moderate discriminant loadings (in decreasing order of magnitude) for SCR-R (*r* = 0.548), SCR-L (*r* = 0.520), SLF-R (*r* = 0.468), PCR-L (*r* = 0.462), PLIC-R (*r* = 0.452), CNG-R (*r* = 0.424), PCR-R (*r* = 0.420), PLIC-L (*r* = 0.415), SLF-L (*r* = 0.401), CC body (*r* = 0.360), CNG-L (*r* = 0.320), PTR-L (*r* = 0.319), whereas function 2 was characterized by UF-R (*r* = 0.409), and CC genu (*r* = 0.338). The resultant discriminant function analysis showed an overall correct classification rate of 96.0% with a cross-validation (“leave-one-out”) technique confirming the stability of this classification procedure with an overall correct rate of 81.8%.

### Multinomial Logistic Regression Models

Given the discriminant function analysis findings, AD and RD scores from the SCR-R, SCR-L, SLF-R and PCR-L, were used for the multinomial logistic regression analysis, as these were revealed to be the 4 most distinguishing tracts according to the discriminant function analysis. The resultant model was found to be significant (- 2 Log Likelihood – 68.10, χ^2^(22) = 139.27, *p* < 0.001) and accounted for 86.1% of the variance (Nagelkerke R^2^ = 0.861). As cluster 1 had the intermediate profile of FA scores, this cluster was chosen to be the reference group. The odds ratio for each variable revealed that, compared to cluster 1, cluster 2 was significantly more likely to have increased RD in the PCR-L, SCR-R and SLF-R (*ps* ≤ 0.040), and decreased AD in the PCR-L (*p* = 0.024), whereas cluster 3 was significantly more likely to have decreased RD in the SCR-R (*p* = 0.011), and increased AD in the SCR-R (*p* = 0.021),

### Correlations

**Fig. 2:**
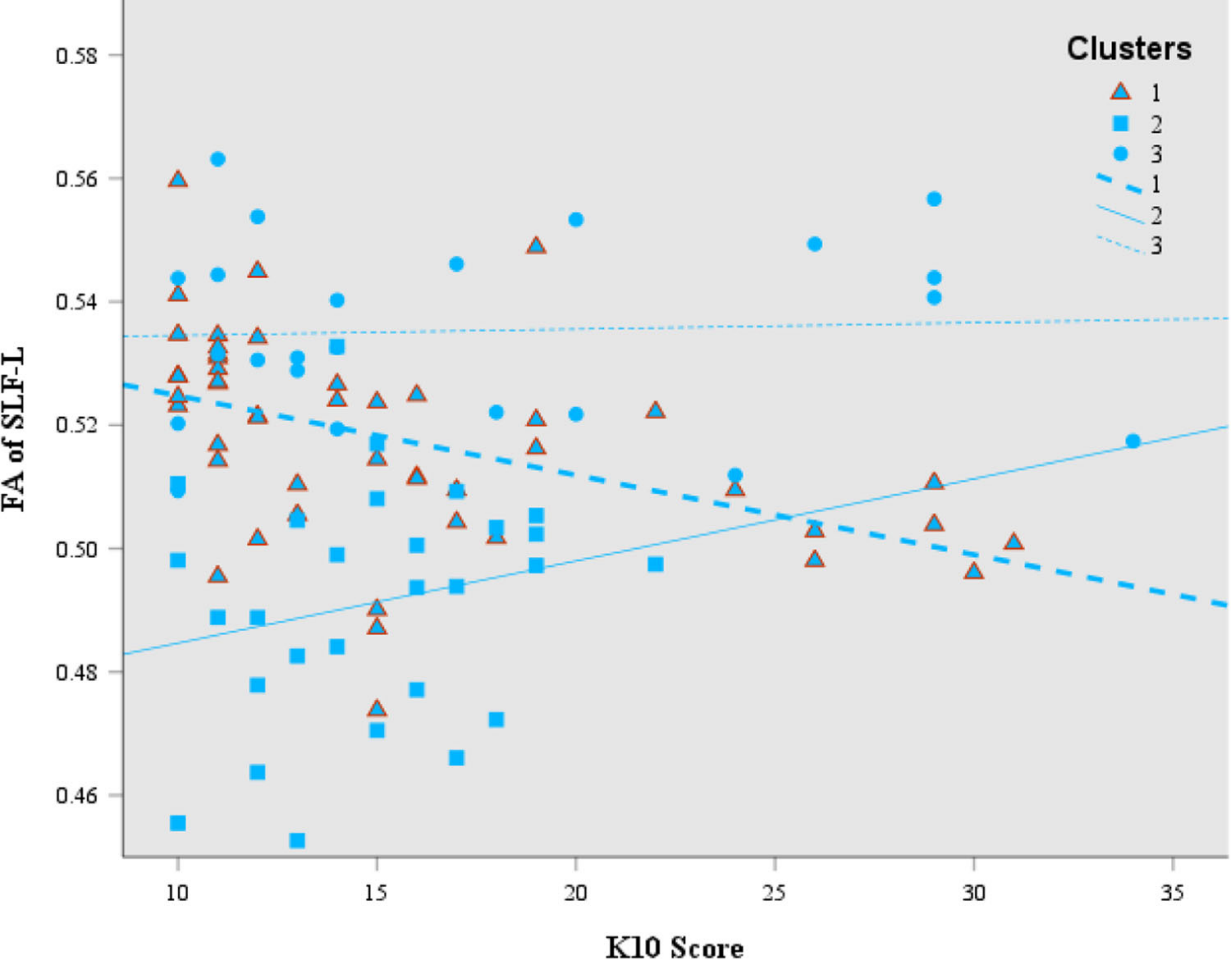
Correlation between FA of the SLF-L and K10 scores in cluster 1 (*r* = −0.462, *p* < 0.001; *N* = 48; triangles), compared to cluster 2 (no significant correlation; *N* = 28; squares) and cluster 3 (no significant correlation; *N* = 23; circles). FA fractional anisotropy; SLF–L superior longitudinal fasciculus, left; K10 scores = psychological distress, with higher scores meaning higher psychological distress

**Fig. 3:**
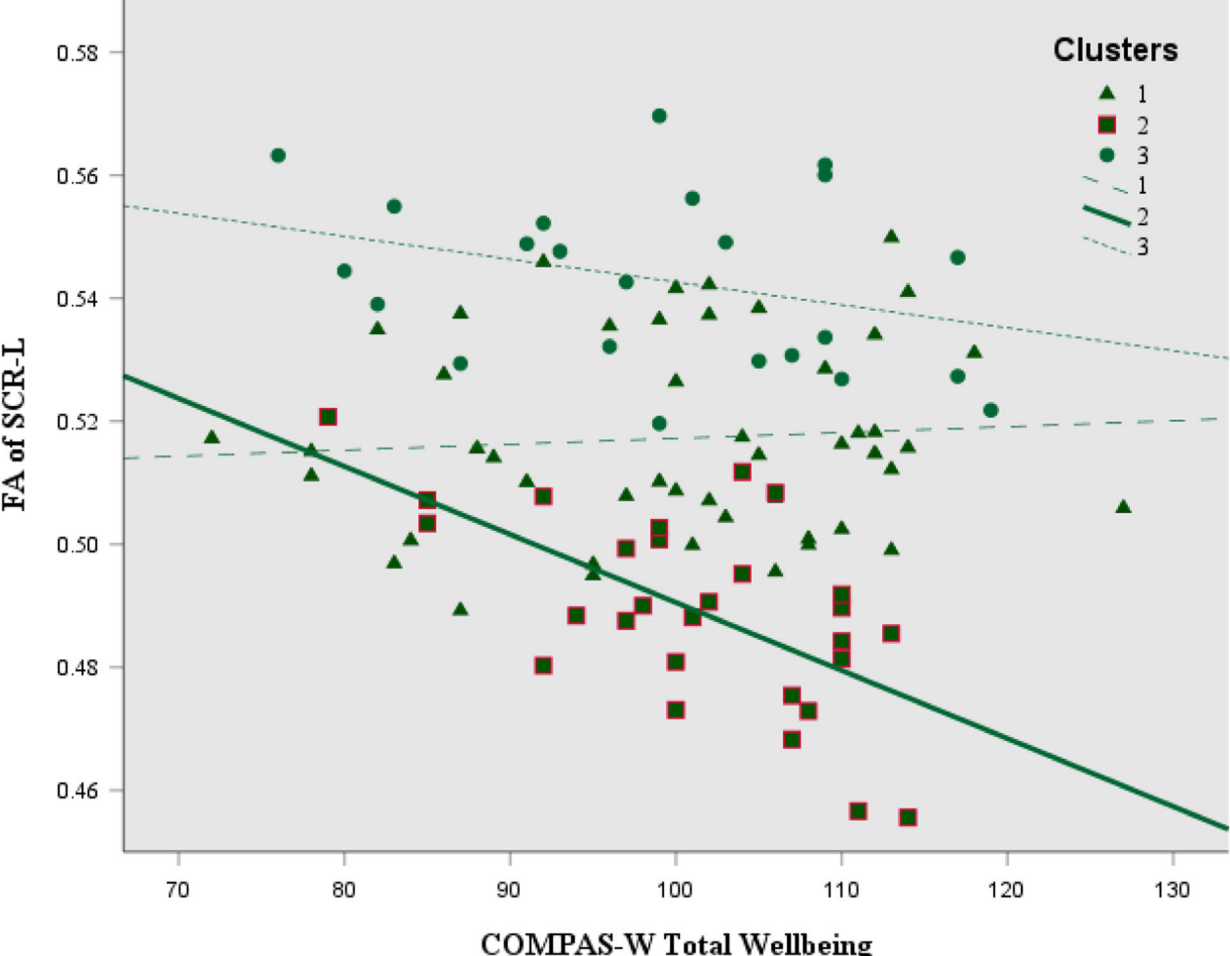
Correlation between FA of the SCR-L and COMPAS-W total wellbeing scores in cluster 2 (*r* = −0.623, *p* < 0.001, *N* = 28; squares), compared to cluster 1 (no significant correlation; *N* = 48; triangles) and cluster 3 (no significant correlation; *N* = 23; circles). FA fractional anisotropy; SCR–L superior corona radiata, left; COMPAS-W Total Wellbeing, with higher scores meaning higher total wellbeing

There were no significant correlations between FA scores of each of the 21 tracts and the self-report variables in the whole sample (*p* < 0.0016). For cluster 1, one significant association was revealed: K10 total correlated with FA in the SLF-L (*r* = −0.462, *p* < 0.001; Fig. 2). For cluster 2, COMPAS-W total wellbeing scores were associated with FA in the SCR-L (*r* = −0.623, *p* < 0.001; Fig. 3). For cluster 3, no significant within cluster correlations were found (*p* < 0.0016).

## Discussion

Previous findings across DTI studies exploring the neurobiology of mental wellbeing in adolescent populations have been heterogenous, with much of the previous research using either a measure of subjective wellbeing or psychological wellbeing, but not both, and had not considered a dual-factor perspective of wellbeing and psychopathology. Therefore, the goal of the current study utilizing DTI data obtained from the First Hundred Brains cohort, a fixed dataset of 101 unique early adolescents, was to identify potential neurobiological profiles from FA values across 21 major white matter tracts throughout the brain, that may underlie differences in measures of wellbeing. Via cluster analysis three distinct and significantly different cluster profiles were identified, and confirmatory discriminant function analysis revealed that these three cluster groups were maximally separated by two key patterns (discriminant functions). First, consistent FA changes in 12 tracts (representing projection, association and commissural fibers); and second, consistent FA changes in the right UF and the CC genu, which are two tracts with functional connections within the frontal lobes. Between-groups analyses revealed that the three clusters did not significantly differ in any measures of mental health and wellbeing. However, two significant left lateralized, negative associations (SLF-L and K10 in cluster 1, and SCR-L and COMPAS-W total in cluster 2) were found, providing preliminary evidence of the potential laterality of the relationship between markers of white matter integrity and mental health and wellbeing specifically in early adolescents.

### Structural Connectivity Differences in Cluster Groups

Overall, the clusters identified were neurobiologically distinct in a few ways. Cluster 1 showed an intermediate FA profile in all tracts compared to clusters 2 and 3, had significantly increased AD scores in the CNG-R and PLIC-L compared to cluster 2, and significantly decreased RD scores in the 14 of the 21 tracts compared to cluster 2 and 3. Although cluster 1 had the intermediate FA profile, such FA values (i.e., increased relative to cluster 2) in combination with increased AD and decreased RD, could be reflective of larger axonal diameter or higher myelination (Feldman et al., 2010).

Cluster 2 had a distinct profile with significantly decreased FA scores across all tracts compared to the other clusters, had significantly increased RD scores in 14 of the tracts compared to the other clusters, and significantly decreased AD scores compared to cluster 1 and 3 in the CNG-R, in the CNG-L compared to cluster 3, and in the PLIC-L compared to cluster 1 and 3. Focusing on the four most distinguishing tracts in terms of FA that were revealed from the discriminant function analysis, the findings from the multinomial logistic regression showed that cluster 2 was significantly more likely to have increased RD scores in PCR-L, SCR-R and SLF-R, and decreased AD in PCR-L compared to cluster 1. Therefore, in the PCR-L specifically, as AD may be a marker of axon numbers and axonal coherence, and RD may represent changes in myelination, axonal packing or myelin integrity (Lebel et al., 2019). The combination of decreased FA, increased RD and decreased AD compared to the other clusters, may be reflective of axonal degeneration, or potentially slower development Feldman et al. (2010).

Cluster 3 had a distinct profile of increased FA scores across all tracts compared to the other clusters and was significantly different across all tracts except for the CST-R and L, UF-R and L, and the CC genu (no different from cluster 1). There were no significant differences in terms of AD scores in cluster 3 compared to the other clusters, but significantly decreased RD scores in 18 tracts compared to cluster 2 were evident. Relating to the multinomial logistic regression, cluster 3 was significantly more likely to have decreased RD and increased AD in the SCR-R specifically. Studies in healthy children suggest that FA and RD values represent axonal packing and diameter, and/or myelination (Krogsrud et al., 2016), therefore, the neurobiological profile of cluster 3 with *increased* FA along with *decreased* RD and unaffected or increased AD, compared to the other clusters, might indicate dense axonal packing, large axonal diameter, excessive myelination, and/or reduced neural branching and functionality of the myelin sheath (Feldman et al., 2010; Soares et al., 2013).

### Mental Health and Wellbeing Across Clusters

While the analysis found no significant differences between the clusters in terms of mental health and wellbeing, there were ostensible health and wellbeing differences across the clusters. For example, cluster 3 had the lowest total quality of life and wellbeing scores (including all subjective wellbeing subscale scores and two out of the three psychological wellbeing subscales), and the highest psychological distress scores. Further, overall cluster 3 had the highest proportion of those categorized as having high or very high psychological distress, and the lowest proportion of those classified as well (according to ABS cut offs). These results taken together with the neurobiological profile of cluster 3, could indicate that dense axonal packing, large axonal diameter, excessive myelination, and/or reduced neural branching and functionality of the myelin sheath in early adolescence may be linked to lower overall mental health and wellbeing. Such patterns may indicate the possible emergence of psychopathology and should therefore be followed longitudinally in this specific cohort to track further potential changes in structural connectivity. In contrast, cluster 2 had the highest overall wellbeing and quality of life scores, and lowest psychological distress scores, with a neurobiological profile of lower FA values. The lack of statistical significance despite ostensible differences in health and wellbeing (particularly in cluster 2 and 3), may in part be due to the small size of the two clusters, and the general population sample as opposed to a clinical sample. Therefore, future research should aim to confirm or refute these trends with larger sample sizes and examine the differences in neurobiological profiles and mental health and wellbeing in more detail, and longitudinally, to establish any potential causal links.

### Associations Between Mental Health and Wellbeing in Individual Clusters

Among all participants (i.e., the whole sample) after a Bonferroni correction, no significant associations with wellbeing measures and FA values were evident. In a previous study from the LABS a consistent pattern of significant correlations in the whole sample (*N* = 73) between social connectedness and FA (negative), RD (positive) and AD (positive) clusters in numerous tracts, including clusters of the CC genu were found (Driver et al., 2023). This indicated that adolescents with lower social connectedness had a white matter profile suggestive of reduced axonal density or coherence. Such consistent association patterns were not evident in the whole sample analysed in the current study.

Despite analyses identifying no significant differences between the three cluster groups in measures of wellbeing, correlation analyses performed on the wellbeing measures and FA values in the individual cluster groups identified two significant associations. Given the seemingly contrasting findings of these two associations, it is important to consider such findings according to the differing subgroup white matter profiles. Cluster 1, with the intermediate white matter profile which may be reflective of larger axonal diameter or higher myelination, had a negative relationship identified for K10 and the SLF-L, such that those with lower psychological distress had higher FA values in this tract. The SLF is one of the largest association fiber bundle systems, connecting frontal, temporal, and parietal areas ipsilaterally (Janelle et al., 2022), and is thought to be responsible for language function, motor planning (left hemisphere), and spatial orientation (mainly right hemisphere) (Janelle et al., 2022), and increases in FA in the SLF has been found to correlate with improved emotion recognition (Mürner-Lavanchy et al., 2020). This tract is said to mature rapidly during adolescence but also shows a protracted development (Lebel et al., 2019). When considering the neurobiological profile of cluster 1 in comparison to cluster 2 (Fig. 2; with the overall white matter profile suggesting axonal degeneration or more likely slower development), this pattern of DTI measures may be reflective of more rapid development in this tract for cluster 1, and the development of the SLF-L may also be linked to better mental health and wellbeing.

The second negative correlation was in cluster 2, between COMPAS-W total wellbeing and the SCR-L, such that those with higher total wellbeing had lower FA values in this tract. This suggests that for this subgroup, with an overall profile of decreased FA (reflecting axonal degeneration or slower development), better wellbeing was associated with lower FA values specifically in the SCR-L. Vitolo et al. (2022) reported that those who were identified as low dispositional users of reappraisal had increased mean diffusivity in the SCR. Although mean diffusivity is based on the averaged diffusion along all axes, higher mean diffusivity values and lower FA values both correspond with less diffusion restriction and less directionality. Therefore, the correlations observed in SCR-L FA in cluster 2, contrast with Vitolo et al’s. (2022) increased mean diffusivity findings, when considering positive markers of wellbeing (i.e., better reappraisal skills and better mental wellbeing). The difference in findings may be due to the broader age range (18–40 years), and mostly female sample, of Vitolo et al’s study, and therefore may be explained by developmental differences. The findings from the current study also contrast with earlier studies that reported that adolescents with depression had lower FA values in the SCR (LeWinn et al., 2014). The SCR is a key projection fiber which connects the brain stem to the cortex and has been found to be associated with various aspects of attention (Stave et al., 2017), arousal, emotional conditioning, and memory consolidation. Additionally, it has been reported that increased wellbeing may be associated with reduced gray matter volume in the pontine nuclei (Gatt et al., 2018), and the authors postulated that the aforementioned processes that involve the brain stem, may therefore play a role in wellbeing, which may explain the association with wellbeing in this brain area and associated white matter tracts. Additionally, it has been reported that wellbeing is not stable in adolescence and may decrease as youth mature (Patalay & Fitzsimons, 2018) and therefore, the higher wellbeing scores in cluster 2 with the corresponding lower FA values may be reflective of slower developmental maturity.

Taken together, the findings of lower FA being associated with higher total wellbeing (cluster 2), and lower FA being associated with higher psychological distress (cluster 1), may also provide collective evidence of psychopathology and wellbeing operating on a functionally independent dual continuum. This further supports the dual-factor models’ emphasis on the need to measure both psychopathology *and* wellbeing for understanding and interpreting levels of the other. Thus, although there were no significant differences between the three cluster groups and the different measures of wellbeing, ignoring the potential for differences in structural connectivity when evaluating relationships with wellbeing measures, would have resulted in an omission of these cluster specific relationships.

Due to both associations being found in left hemisphere tracts, there appears to be a slight left side dominance in the current study, suggesting that FA values in left hemisphere tracts may be more representative as neurobiological markers of mental health and wellbeing outcomes in early adolescence. In previous LABS findings via electroencephalography, there were hemispheric differences in relationships with neural activity and psychological distress and wellbeing (Sacks et al., 2023). Similar to the current study, a significant left lateralized relationship was found with wellbeing, however a right lateralized relationship was found with psychological distress (Sacks et al., 2023). The lateralized relationships were postulated to potentially provide support for a dual factor model of mental health and wellbeing (Sacks et al., 2023). In a an earlier review of neuroimaging studies however, it was concluded that although there was some evidence to suggest left sided laterality in regards to the dorsolateral prefrontal cortex and positive affect, there was overall limited literature to definitively conclude evidence of laterality of any associations with the brain and mental health and wellbeing (King, 2019). Relating to white matter specifically, previous literature finding associations between aspects of wellbeing and structural connectivity, only reported on bilateral findings (Jung et al., 2022; Vitolo et al., 2022). Additionally, although Kotikalapudi et al. (2022) reported that optimism was associated with AD in numerous tracts in the right hemisphere exclusively, their participants were mostly female and ranged from 18 to 40 years old. From a developmental perspective, a recent study reported on the FA values of the three branches of the SLF, finding a significant right lateralization in adolescents, but only in two portions of the SLF (Amemiya et al., 2021), whilst a longitudinal study in 9–13 year olds reported that pubertal stage was positively correlated with fiber density in the SLF-R specifically (Genc et al., 2020). Another study following female participants annually for six years from age nine, found that earlier pubertal timing (but not tempo), predicted greater FA and in left-lateralized tracts, including the corona radiata (Chahal et al., 2018). An earlier small study reported that girls (*n* = 29 aged 12–14 years) exhibited higher FA in the SCR-R compared to aged matched boys, who exhibited higher AD in several tracts including the SLF-R (Bava et al., 2011). Consequently, as there appears to be inconclusive evidence in previous literature relating to lateralization in terms of adolescent development and relating to mental health and wellbeing measures, the findings from the current study provide preliminary evidence for the potential laterality of the relationship between markers of white matter integrity and mental health and wellbeing specifically in early adolescents.

### Limitations

There are some limitations to this study that warrant discussion. Firstly, due to the cross-sectional exploratory nature of this study, the results limit understanding of the potential developmental underlying neurobiological processes. It is acknowledged that a key role of developmental neuroimaging is to elucidate any variability in behavioral and cognitive development within a representative sample (Lebel et al., 2019). As such, the First Hundred Brains cohort is comprised only of 12–13-year old’s, from a general population sample, with the goal of future research with this cohort to expand on these cross-sectional findings with follow up longitudinal research, as this cohort progresses through adolescence. Secondly, as this study was exploratory, future studies should aim to refute or confirm such findings. Next, although there is evidence that stage of puberty may influence white matter development (Genc et al., 2017), this was not measured in the current study. However, the constrained age range in this cohort reduces the likelihood of difference due to puberty, and a puberty measure has since been added to the LABS research program which can be included in future longitudinal follow-up studies with the First Hundred Brains cohort. Regarding the cluster groups, although cluster 2 and 3 were adequate for statistical power *N* ≥ 20;(Dalmaijer et al., 2022), a larger sample size would be beneficial to enhance the statistical power of the subsequent analysis of the cluster groups. However, overall the large sample size within this controlled age bracket far exceeds thresholds that are considered adequate for neuroimaging sample sizes (Vitolo et al., 2022). Further, the small age range of participants in this study reduces the potential for developmental variation and is a strength of the current study compared to previous research that includes much broader age ranges. The selection of white matter tracts in this study was based on previous literature that implicated these tracts in wellbeing related constructs and represented each of the three types of tracts. However, it is possible that the integrity of other white matter tracts that have not been included in this analysis may be associated with wellbeing and may contribute to further differentiation of the cluster groups. Therefore, future research could expand the breadth of tracts investigated to further understanding of the neurobiological markers of wellbeing in adolescents. Finally, it is acknowledged that crossing fibers are an inherent problem in certain DTI software and analysis techniques (Schilling et al., 2022), which can result in lower FA values within a certain voxel (Feldman et al., 2010), and FA values depend significantly on type of acquisition and analysis used (Lebel et al., 2019). Therefore, it is possible that these results have been affected by crossing fibers. Future research including alternative volumetric measures of white matter may provide more evidence for the interpretation of the current findings.

### Implications

The data driven exploratory approach of this study provides preliminary evidence of variations in indicators of white matter integrity in early adolescence that may be linked to differences (albeit subtle in this study), to mental health and wellbeing. In other words, the analysis undertaken here revealed associations across two white matter tracts in clusters with significantly different profiles of DTI measures, that were not observed at the whole sample level. As previous literature is largely varied and somewhat disparate relating to white matter and mental health and wellbeing in early adolescence, the findings from this study contribute to building a more comprehensive understanding of the potential neurobiology of wellbeing in early adolescence. Given the nature of the LABS and that this cohort is sampled from the general population, the white matter profiling and associated mental health and wellbeing measures, although subtle, may be indicative of different levels of both protection and risk of emerging psychopathology, which need to be tracked longitudinally to validate such preliminary evidence. That is, the cluster group with poorer mental health and wellbeing metrics and corresponding perturbations in structural connectivity indicators (cluster 3) may be at risk of developing mental disorders. In a DTI based cluster analysis study of an older clinical population, similar subgroups were found with corresponding associations with mental health outcomes (i.e., severity of symptoms and functioning) (Hermens et al., 2019). Along with the evidence that 50% of mental disorders occur before the age of 14, this supports the notion that identifying the early neurobiological markers of mental health and wellbeing, and emerging psychopathology, and continuing to monitor subgroups from early adolescents, is warranted. Research focused on adolescent mental health vulnerability *and* opportunity, is vitally important for early detection and prevention of psychopathology, and improvement in mental health and wellbeing. Therefore, it is the intention of the LABS to further track such neurobiological markers of mental health and wellbeing using the fixed First Hundred Brains cohort as they progress through adolescence. Additionality, in early adolescence specifically, the dual-factor model of mental health and wellbeing was found to be valuable in observing and interpreting changes in wellbeing and psychopathology profiles over time, whereby it was identified that those with low peer support were most likely to change from complete mental health to vulnerable status (Petersen et al., 2022), Therefore, should the profiles of the current adolescent cohort worsen, then interventions that target modifiable factors such as social connectedness (Driver et al., 2023) and sleep (Jamieson et al., 2020), that can affect white matter integrity perturbations, may be warranted. Improving assessment and characterization of wellbeing, and monitoring changes longitudinally may be key to improving clinical assessments and subsequent interventions for youth.

## Conclusion

Previous research exploring adolescent mental health and wellbeing and associated neurobiological markers has been disparate and limited by broad age ranges and the utilization of individual measures of mental wellbeing, as opposed to a composite measure. By using an exploratory data-driven approach to analyze white matter profiles and mental health and wellbeing measures from a fixed cohort of early adolescents, this study found three distinct profiles reflecting different white matter features across 21 major tracts. While there were there were no significant differences between the three cluster groups and the different measures of wellbeing, the current study provides preliminary evidence in terms of the left lateralization of the associations with wellbeing measures that were found within two of the three neurobiologically different clusters. The current study therefore contributes to the growing knowledge of the neurobiological markers of mental health and wellbeing in early adolescence, and by continuing to follow the First Hundred Brains cohort as they progress through adolescence, the future aim is to track such measures over time, providing more detailed insight into the potential neurobiological markers of mental health and wellbeing.
